# 1,25-hydroxyvitamin D relieves colitis in rats via down-regulation of toll-like receptor 9 expression

**DOI:** 10.3325/cmj.2015.56.515

**Published:** 2015-12

**Authors:** Zhang-han Dai, Bei Tan, Hong Yang, Ou Wang, Jia-ming Qian, Hong Lv

**Affiliations:** 1 Department of Gastroenterology, Peking Union Medical College Hospital, Chinese Academy of Medical Science and Peking Union Medical College, Beijing, China; 2 Division of Gastroenterology and Hepatology, Ren Ji Hospital, School of Medicine, Shanghai Jiao Tong University, Shanghai Institute of Digestive Disease, Shanghai Inflammatory Bowel Disease Research Center, Shanghai, China; 3 Department of Endocrinology, Key Laboratory of Chinese Health Ministry, Peking Union Medical College Hospital, Chinese Academy of Medical Science and Peking Union Medical College, Beijing, China.

## Abstract

**Aim:**

To investigate the therapeutic and immunoregulatory effects of 1,25-dihydroxyvitamin D (1,25(OH)D_3_) on 2,4,6-trinitrobenzenesulfonic acid (TNBS) -induced colitis in rats.

**Methods:**

Experimental colitis induced by enema administration of TNBS plus ethanol was treated with 5-aminosalicylic acid (5-ASA) and/or 1,25(OH)D_3_. Disease activity was measured using the disease activation index (DAI), colon macroscopic damage index (CMDI), histological colonic damage score, and myeloperoxidase (MPO) activity. The expression of toll-like receptor 9 (TLR9) in the colon was determined by reverse transcription-polymerase chain reaction and immunohistochemistry.

**Results:**

Rats with TNBS-induced colitis had significantly elevated DAI, CMDI, histological colonic damage score, and MPO activity (all *P* < 0.001) compared to rats without colitis. Treatment with 5-ASA or 1,25(OH)D_3_ ameliorated colitis by lowering CMDI (*P* = 0.049, *P* = 0.040, respectively), histological colonic damage score (*P* = 0.010, *P* = 0.005, respectively), and MPO activity (*P* = 0.0003, *P* = 0.0013, respectively) compared with the TNBS group. Combined treatment with 5-ASA and 1,25(OH)D_3_ significantly decreased MPO activity (*P* = 0.003). 1,25(OH)D_3_ attenuated colitis without causing hypercalcemia or renal insufficiency. TNBS significantly increased the number of TLR9 positive cells compared to control (*P* < 0.010), while 5-ASA, 1,25(OH)D3, and combined treatment with 5-ASA and 1,25(OH)D3 significantly decreased it compared to TNBS group (all *P* < 0.010). In TNBS group a moderate correlation was observed between MPO activity and the number of TLR9-positive cells (r = 0.654, *P* < 0.001).

**Conclusion:**

TLR9 expression correlates with the extent of inflammation in TNBS-induced colitis. 1,25(OH)D_3_ relieves this inflammation possibly by decreasing TLR9 expression.

Vitamin D is a well-known endocrine regulator of calcium homeostasis and 1,25-dihydroxyvitamin D (1,25(OH)D_3_) is its biologically active form. Recent studies suggest that vitamin D deficiency is associated with the onset or increased activity of autoimmune diseases, especially Th1-mediated autoimmune diseases, such as rheumatoid arthritis, systemic lupus erythematosus, diabetes, multiple sclerosis, and inflammatory bowel disease (IBD) (1-3). Furthermore, in Chinese patients with IBD, vitamin D deficiency was observed to be partially related to higher risk of osteoporosis and increased disease activity (4). Therefore, vitamin D deficiency is considered an environment risk factor of IBD. Some researchers have proposed that vitamin D can modulate intestinal microflora, inhibit the adhesion of intestinal bacteria (5), protect the intestinal mucosal barrier (6,7), and reduce excessive external antigens presentation by antigen presenting cells (APCs) (8,9).

The most important antigen recognition receptors on APCs are toll-like receptors (TLRs). The TLRs signaling pathway is thought to play a key role in both innate immunity and adaptive immunity (10). TLRs recognize diverse ligands, including lipids, lipoproteins, proteins, and nucleic acids derived from microbes. TLR9 recognizes unmethylated cytosine-phosphate-guanine (CpG) DNA motifs in bacteria and DNA viruses (11) and is essential for maturation of dendritic cells and release of pro-inflammatory cytokines. Increase in the expression of TLR9 was related to exposure of colonic epithelial cells to pathogenic bacterial DNA (12). Some reports suggested that the excessive inflammatory response associated with a TLR9 polymorphism correlated with an increased risk of Crohn’s disease (CD) (13,14).

Previous studies have increased our understanding of the crosstalk between vitamin D and TLRs. Dickie et al (15) demonstrated that 1,25(OH)D_3_ down-regulated intracellular TLR9 expression and TLR9-induced IL-6 production in healthy human monocytes in vitro. It was also shown that by down-regulating the expressions of TLRs and pro-inflammatory cytokines it improved herpes simplex virus-induced Behcet’s disease-like symptoms in mice (16). On the other hand, Edfeldt et al (17) found that TLRs enhanced bioconversion of 25-hydroxyvitamin D_3_ to its active metabolite, 1,25(OH)D_3,_ by inducting 25-hydroxyvitamin D-1α-hydroxylase_._ Although previous studies showed that vitamin D could modulate intestinal microflora and TLR9 expression, the effect of vitamin D on IBD and its relationship with TLR9 expression in vivo still remained unclear. Therefore, the aim of this study was to investigate the effect of 1,25(OH)D_3_ on 2,4,6-trinitrobenzenesulfonic acid (TNBS)-induced colitis and TLR9 expression in rats.

## Materials and methods

### Rats experiments and induction of colitis by TNBS

Male, 8-10 week old Sprague-Dawley rats, weighing approximately 220 g, were obtained from Animal Science Department of Peking University Health Science Center (Beijing, China). All studies were approved by the Ethics Committee of Peking Union Medical College and were in agreement with the Beijing laboratory animal management guidelines.

TNBS (100 mg/kg, Sigma-Aldrich, Shanghai, China) and 50% ethanol were administered to anesthetized rats through a 2 mm polyethylene tube, which was carefully inserted into the rat’s rectum. To ensure uniform distribution of TNBS throughout the entire colon, rats were held in a vertical position for 5 min after the instillation of the TNBS enema.

1,25(OH)D_3_ (10 μg, Sigma-Aldrich) was dissolved in 4 mL ethanol and diluted with olive oil to obtain the concentration of 0.1 μg/mL. 1,25(OH)D_3_ and 5-ASA (Dr Falk Pharma GmbH, Freiburg, Germany) was administered by gavage. Thirty rats were randomly divided into five groups. One group was treated with ethanol only and served as control group. One group was treated with TNBS only. Vitamin D treatment rats received 0.2 μg/kg/d 1,25(OH)D_3_. 5-ASA treatment rats received 0.4 g/kg/d 5-ASA. Combined 1,25(OH)D_3_ and 5-ASA treatment rats received 0.2 μg/kg/d 1,25(OH)D_3_ and 0.4g/kg/d 5-ASA. These treatments were initiated one day after the instillation of the TNBS enema and continued for 9 days. On the day 10, the rats were sacrificed and their colons removed. To monitor the adverse reaction of 1,25(OH)D_3,_ serum calcium levels and serum creatinine levels were determined at the end of the experiment.

### Assessment of inflammatory activity

*Disease Activation Index (DAI)*. DAI was used to assess the clinical severity of colitis according to Murano et al (18). The calculation was based on the daily body weight, stool consistency, and rectal bleeding. The loss of body weight was scored as follows: 0, no weight loss; 1, weight loss of 0 to 5%; 2, weight loss of 5 to 10%; 3, weight loss of 10 to 20%; and 4, weight loss >20%. Stool consistency was scored as follows: 0, normally formed pellets; 2, pasty and semiformed pellets; and 4, liquid stool. Rectal bleeding was scored as follows: 0, no blood from the rectum and 4, gross bleeding from the rectum. These scores were summed, resulting in a total clinical score ranging from 0 to 12.

*Colon Macroscopic Damage Index (CMDI)*. The assessment of the CMDI was based on the area of inflammation and the presence of ulcers, according to Wallace and Keenan (19). Features were graded as follows: 0, no ulcer, no inflammation; 1, no ulcer, local hyperemia; 2, ulceration without hyperemia; 3, ulceration and inflammation at one site only; 4, two or more sites of ulceration and inflammation; 5, ulceration extending more than 1 cm; 6, ulceration extending more than 2 cm.

*Histological colonic damage*. For histological examination, a sample of colonic tissue located 4-5 cm above the anal margin was obtained from rats in all treatment groups. The sections were stained with hematoxylin and eosin using routine techniques. Histological colonic damage was scored according to Sykes criteria (20). Features were graded from 0 to 5 in a blinded fashion, as follows: 0, no significant damage; 1, damage confined to the epithelium; 2, focal ulcers, limited to the mucosa; 3, focal transmural inflammation and ulceration; 4, extensive transmural inflammation and ulceration with normal mucosa around; 5, extensive transmural inflammation and ulceration with no normal mucosa.

*Measurement of myeloperoxidase (MPO) activity*. The severity of acute colitis was measured by infiltration of neutrophils determined by photometrical assay for the neutrophil-specific MPO enzyme. The MPO activity assay was performed using an MPO kit (Jiancheng Bioengineering Institute, Nanjing, China) according to the method described by Bradley et al (21). The enzyme activity was determined photometrically. The MPO-catalyzed redox reaction of 3,3′-dimethoxybenzidine changed the absorbance at 460 nm. The values were expressed as MPO units per gram of wet tissue.

### Reverse transcription-polymerase chain reaction (RT-PCR) of TLR9 mitochondrial RNA (mRNA)

The expression of the *TLR9* gene at the mRNA level in samples of rats’ colons was measured by RT-PCR. This method enables detection and semi-quantification of gene expression. RNA was extracted from samples of colonic tissue located 5-6 cm above the anal margin using an RNAprep pure Tissue Kit (Tiangen Biotech, Beijing, China). RNA (2 μg) was used as a template in a cDNA synthesis reaction performed in a Quant cDNA kit (Tiangen Biotech, Beijing, China). Housekeeping gene β-actin was amplified as reference gene for mRNA quantification. The primers for TLR9 were forward 5′- TCAACAAGAACACGCTCAGG-3′ and reverse 5′-GAGAGCTGGGGTGAGACTTG-3′ and β-actin forward 5′ -TCCTGTGGCATCCATGAAACT-3′ and reverse 5′-GAAGCATTTGCGGTGCACGAT-3′. The primers were synthesized by SinoGenoMax (Beijing, China). Amplifications were carried out in an Eppendorf gradient thermal cycler (Eppendorf, Hamburg, Germany). PCR cycling consisted of initial denaturation at 94°C for 3 min, followed by 35 cycles at 94°C for 30 s, 55°C for 30 s, and 72°C for 1 min, and then maintained at 72°C for 5 min. 10 μL of the PCR reaction was run on precast 2.5% agarose Tris/Boric Acid/EDTA gels at 120 V. The level of TLR9 transcripts was calculated by relative quantification (22). The RT-PCR results were expressed as the level of TLR9 transcripts relative to the level of β-actin transcripts in the same samples using Quantity One 4.61 (Bio-Rad, Hercules, CA, USA).

### Immunohistochemistry (IHC) of TLR9 protein

IHC was performed using a specific primary mouse monoclonal antibody (Abcam, ab12121, Cambridge, UK) against TLR9 in a final 1:50 dilution. First, deparaffinized sections were heated in citrate buffer (pH 6.0) to induce epitope retrieval. After slow cooling to room temperature, the slides were incubated in 10% hydrogen peroxide and goat serum to block unwanted epitopes. The slides were then washed in phosphate-buffered saline (PBS) twice for 5 min and incubated overnight at 4°C with primary antibody. Following this, they were stained with a chromogen purchased from ZSGB-BIO (PV-9000) (Beijing, China), washed in PBS, and counterstained with hematoxylin. The specimens were processed in the absence of primary antibody as negative controls. Positive staining was defined microscopically by visual identification of brown pigmentation either in the cytoplasm or nucleus. The TLR9 positive cells were counted in 10 high power fields of each lesion by light microscopy at ×400 magnification. The number of TLR9 positive cells in each lesion was determined as the average number in 10 high power fields.

### Statistical analysis

Statistical analysis was performed using GraphPad Prism 5 statistical software (GraphPad Software, Los Angeles, CA, USA). Normality of distribution was tested using Shapiro-Wilk test and the data are expressed as mean ± standard deviation (SD). The differences between groups were tested using one-way analysis of variance and least-significant difference test. Correlation analysis was performed by bivariate correlation. Pearson correlation coefficient (r)>0.95 indicated significant correlation; r ≥0.8 high correlation; 0.5≤r <0.8 moderate correlation; 0.3≤r <0.5 low correlation; r <0.3 no correlation; -1≤r <0 negative correlation. Differences were considered statistically significant at *P* < 0.050.

## Results

### TNBS induced acute colitis in rats

**Figure 1:**
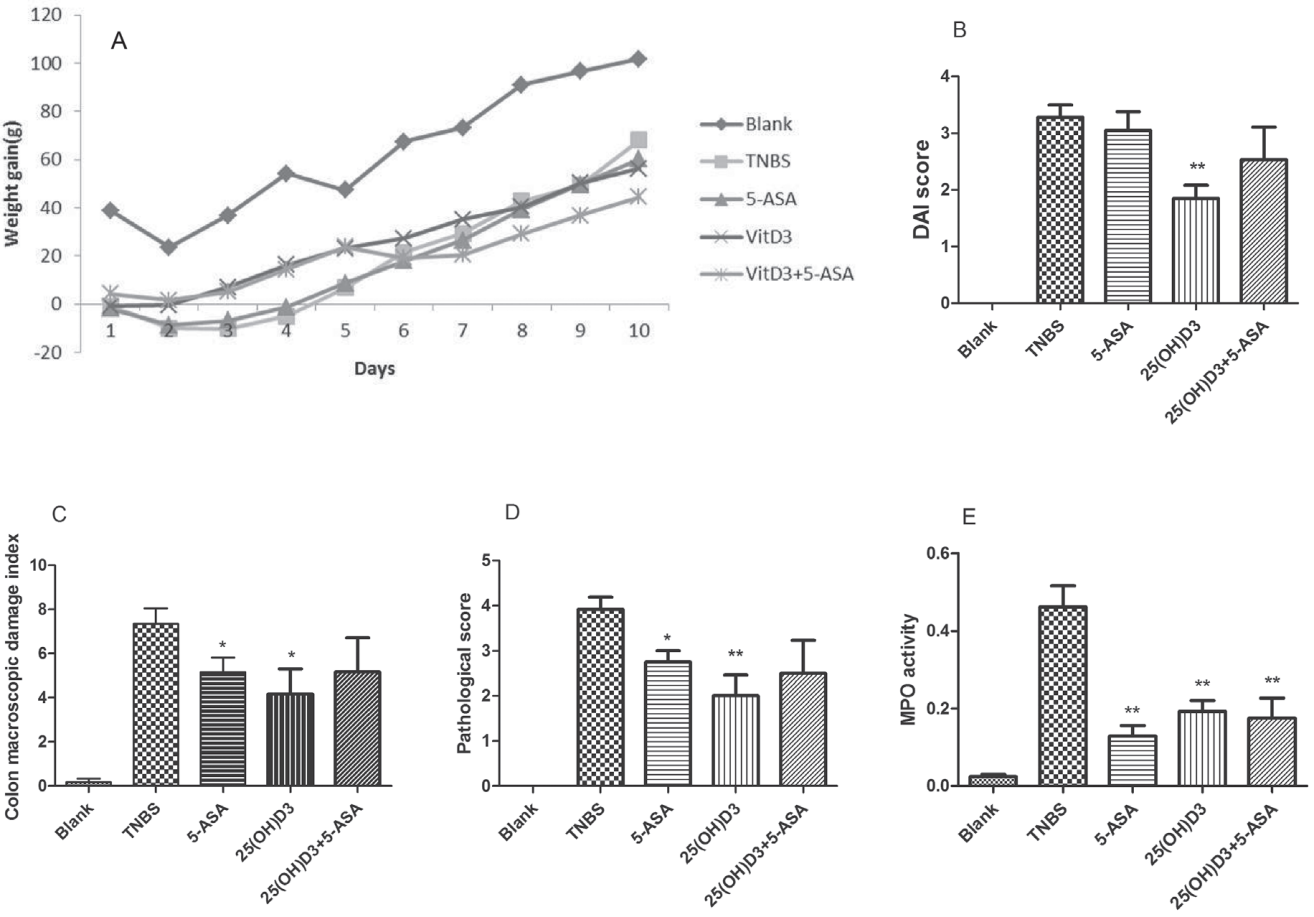
Analysis of colitis severity in rats under different treatments. (**A**) Weight gain. (**B**) Disease activation index score. (**C**) Colon macroscopic damage index. (**D**) Pathological score. (**E**) Myeloperoxidase activity. TNBS, 2,4,6-trinitrobenzenesulfonic acid; 5-ASA, 5-aminosalicylic acid; VitD3, 1,25-dihydroxyvitamin D. Data are presented as mean ± standard deviation (n = 6). *P < 0.050; **P < 0.010 vs TNBS-treated rats.

**Figure 2:**
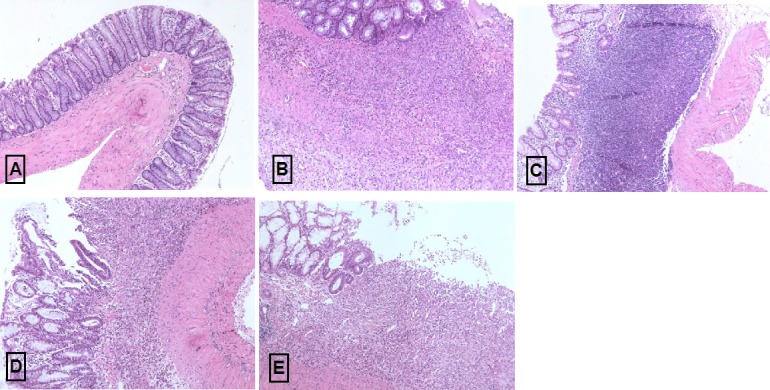
Microscopic features of colons. (**A**) Control group; (**B**) TNBS-treated group; (**C**) TNBS+ 5-ASA treated group; (**D**) TNBS+ VitD3 treated group; (**E**) TNBS+5-ASA+ VitD3 treated group. The sections were stained with hematoxylin and eosin. Magnification: 200 × . TNBS, 2,4,6-trinitrobenzenesulfonic acid; 5-ASA, 5-aminosalicylic acid; VitD3, 1,25-dihydroxyvitamin D.

Rats treated with TNBS developed acute colitis, characterized by weight loss, diarrhea, and rectal bleeding, especially in the first three days. Their body weight increased from the day 4 (Figure 1A). On the day 10, the control group had significantly greater weight than other groups (all *P* < 0.001); while there was no difference between TNBS-treated groups (*P* = 0.937, *P* = 0.571, *P* = 0.941) (Figure 1A). TNBS-treated rats had significantly higher DAI, CMDI, pathological score, and MPO values than control rats (all *P* < 0.001) (Figure 1B-E). In gross specimens, TNBS colitis is characterized by severe hyperemia and focal mucosal necrosis. In the TNBS colitis groups, transmural inflammation was observed, characterized by infiltration of inflammatory cells in the lamina propria. Some HE sections of TNBS-treated colitis groups showed ulcerations, loss of goblet cells, and diffuse fibrosis. Focal granulomas were also observed (Figure 2B-E).

### 5-ASA and 1,25(OH)D_3_ attenuate TNBS colitis

We then treated TNBS-induced colitis rats with 5-ASA, 1,25(OH)D_3_ and their combination. 5-ASA-treated rats showed significantly decreased CDMI (*P* = 0.049), pathological score (*P* = 0.010), and MPO activity (*P* < 0.001). 5-ASA treatment also restored normal histological appearance of the samples (Figure 2C). 1,25(OH)D_3_-treated rats also showed significantly decreased DAI value (*P* = 0.001), CDMI (*P* = 0.040), pathological score (*P* = 0.005), and MPO activity (*P* = 0.005). 1,25(OH)D_3_ treatment diminished the intensity of inflammatory infiltration in the lamina propria (Figure 2D).

Rats treated with both 5-ASA and 1,25(OH)D_3_ showed significantly decreased MPO activity (*P* = 0.003), but the difference in pathological score and MPO activity was not significant (*P* = 0.099) (Figure 1D-E). Thus, the combined treatment did not induce a synergistic effect.

### Rat blood serum calcium and creatinine profiles

**Figure 3:**
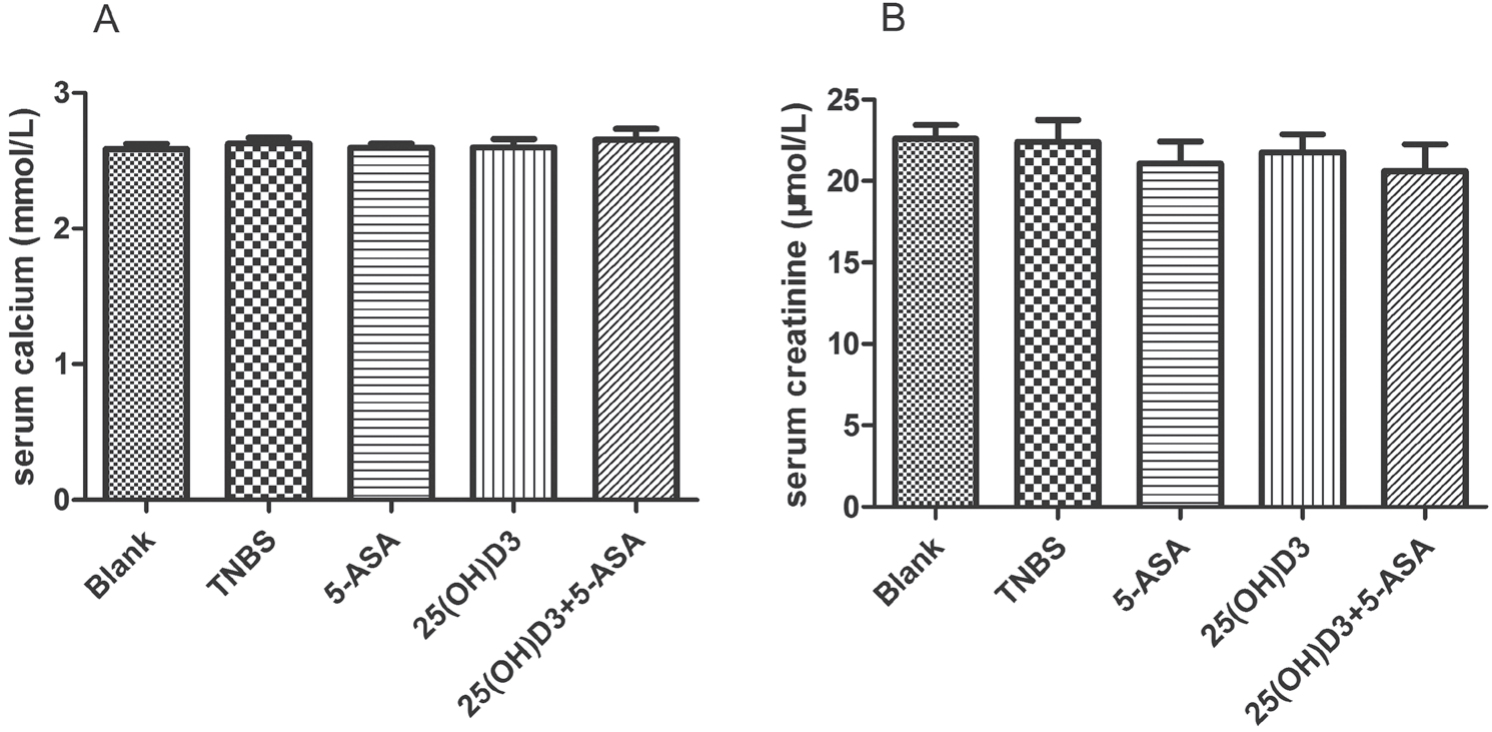
Serum calcium and creatinine profiles. (**A**) serum calcium levels (mmol/L). (**B**) serum creatinine levels (μmol/L). TNBS, 2,4,6-trinitrobenzenesulfonic acid; 5-ASA, 5-aminosalicylic acid; VitD3, 1,25-dihydroxyvitamin D. Data are presented as mean ± standard deviations (n = 6).

To monitor the side effects of 1,25(OH)D_3,_ hypercalcemia and renal insufficiency, we measured serum calcium levels and serum creatinine levels. Treatment with 1,25(OH)D_3_ alone and in combination with 5-ASA did not significantly alter serum calcium and creatinine levels (Figure 3).

### 5-ASA and 1,25(OH)D_3_ down-regulate TLR9 expression and the number of TLR9 positive cells in TNBS-induced colitis

**Figure 4:**
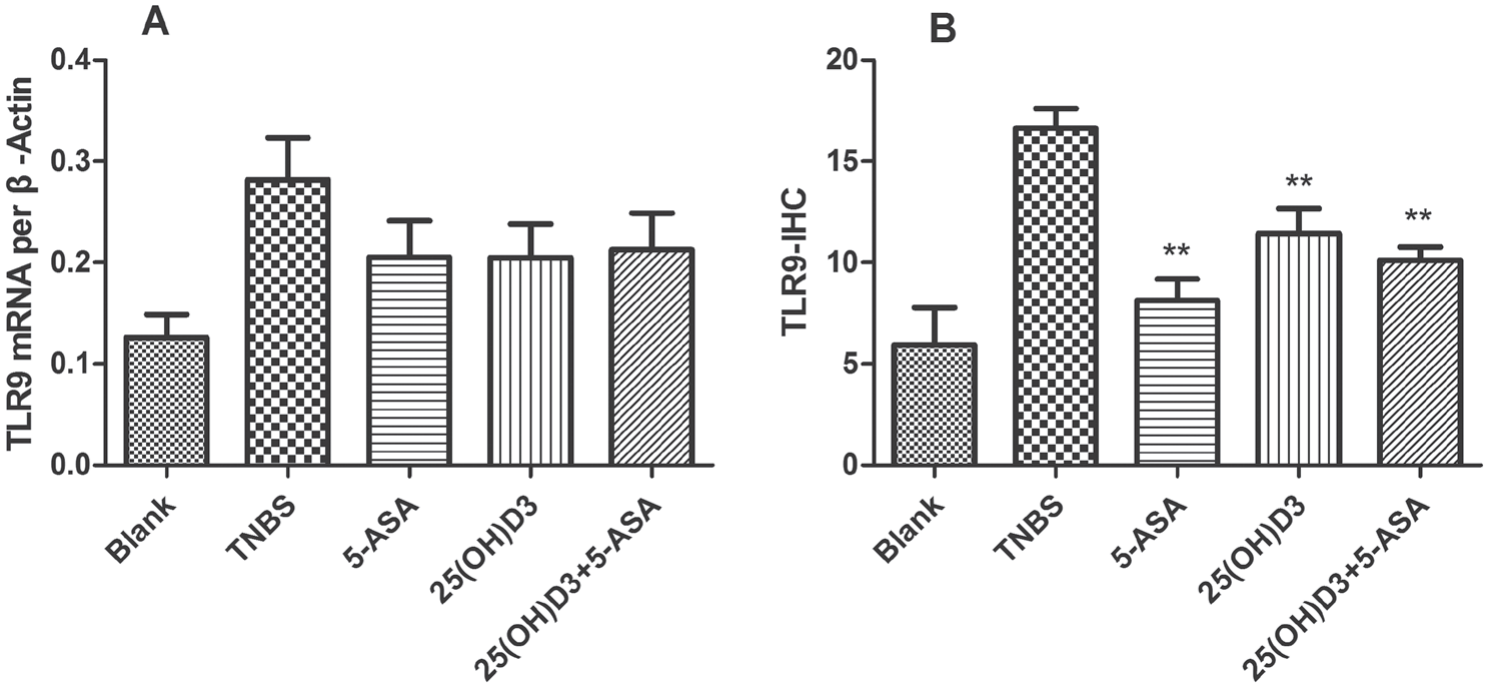
Expression of toll-like receptor 9 (TLR9) in colon. (**A**) Relative expression of TLR9 mRNA level in the colons from each group using β-actin as the internal control gene. (**B**) The number of TLR9 positive cells in colon from each group. (**C**) Schematic diagram of TLR9 activation increases myeloperoxidase activity through nuclear factor kB signaling pathway. TLR, toll-like receptor; TNBS, 2,4,6-trinitrobenzenesulfonic acid; 5-ASA, 5-aminosalicylic acid; VitD3, 1,25-dihydroxyvitamin D; NF-kB, nuclear factor kB; TNF, tumor necrosis factor; IL, interleukin; IFN, interferon; MPO, myeloperoxidase; nVDR, nuclear vitamin D receptor. Data are presented as mean ± standard deviation (n = 6). **P < 0.010 vs TNBS-treated rats.

The mRNA expression of the *TLR9* gene was determined using RT-PCR. TLR9 mRNA expression was significantly higher in the TNBS than in the control group (*P* = 0.008). In 5-ASA-treated rats (*P* = 0.194) and 1,25(OH)D_3_-treated rats (*P* = 0.178) *TLR9* gene expression was reduced compared to TNBS group, but neither reduction was statistically significant (Figure 4A).

**Figure 5:**
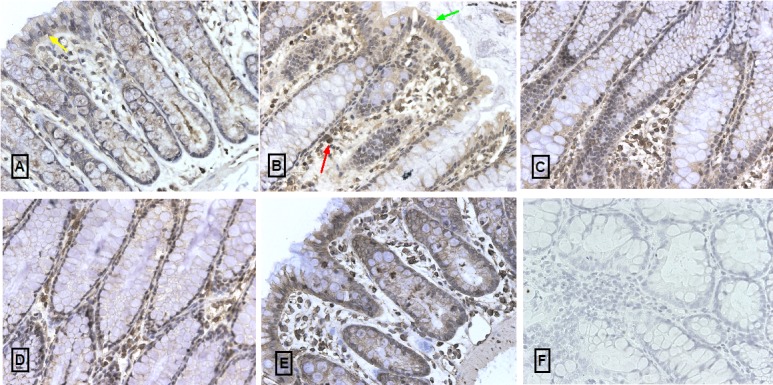
Immunohistochemical staining using an anti-TLR9 primary antibody. (**A**) Control group. (**B**) TNBS group. Cells that expressed TLR9 were mostly unevenly distributed in the lamina propria (red arrow). Some goblet cells and epithelium cells were TLR9-positive (green arrow). (**C**) TNBS+5-ASA group. (**D**) TNBS+ VitD3 group. (**E**) TNBS+5-ASA+ VitD3 group. (**F**) Negative control. Magnification: 400 × . TLR, toll-like receptor; TNBS, 2,4,6-trinitrobenzenesulfonic acid; 5-ASA, 5-aminosalicylic acid; VitD3, 1,25-dihydroxyvitamin D.

We then assessed the number of TLR9-positive cells by IHC. In the control group, only a few inflammatory TLR9-positive cells were present in the epithelium, lamina, and submucosa. In TNBS-treated group, the number of TLR9-positive cells was significantly higher (all *P* < 0.010) and they were mostly distributed in the lamina propria. Some goblet cells and epithelial cells were TLR9 positive (Figure 5A-E). 5-ASA treatment, 1,25(OH)D_3_ treatment, and combined treatment with 5-ASA and 1,25(OH)D_3_ significantly reduced the number of TLR9-positive inflammatory cells (*P* < 0.001, *P* = 0.008, *P* < 0.001, respectively) (Figure 4B). In TNBS-induced colitis group, a moderate correlation was detected between MPO activity and the number of TLR9-positive cells (r = 0.654, *P* < 0.001) and a low non-significant correlation between MPO activity and TLR9 gene expression (r = 0.394, *P* = 0.057).

## Discussion

Consistent with the results of previous research (23,24), our study showed that 1,25(OH)D_3_ reduced inflammation in TNBS-induced colitis by suppressing infiltration of neutrophil granulocytes. However, the combined application of 5-ASA and 1,25(OH)D_3_ was no more effective than treatment with 1,25(OH)D_3_ alone, suggesting no synergistic effects. Vitamin D usage is considered as an adjuvant therapy in IBD, based on previous murine experiments and human studies (25,26). It is possible that the therapeutic effect of vitamin D is not substantial. Also the lack of synergistic effects in our study might reflect an insufficient statistical power of our study. Though numerous studies have reported anti-inflammatory effect of 1,25(OH)D_3_ in vitro, only one placebo-controlled trial evaluated its effect in CD patients (27). Jorgensen et al (27) reported that treatment with vitamin D markedly reduced the rate of clinical relapses from 29% to 13%, but the reduction was not significant. It is possible that 1,25(OH)D_3_ is beneficial to IBD patients, but further large-scale, multi-center clinical trials are needed to investigate this issue.

TLRs are usually expressed in inflammatory cells, such as macrophages and dendritic cells, and play a central role in the innate immune system. TLR9 binds to unmethylated CpG DNA in intestinal bacteria and activates an intestinal immune response through the NF-κB signaling pathway. We demonstrated that a small amount of TLR9 was expressed in the colon of control rats, which is consistent with previous studies (28,29). The limited expression of TLR9 in control rat colons could reduce intestinal commensal bacteria antigen recognition, and help control the specific immune response. In TNBS-induced colitis, a significant increase in TLR9 expression was observed. Consistent with the results of previous studies in IBD patient specimens (30,31) and other animal experiments (32), our data showed that TLR9 was not only expressed in inflammatory cells in rats with colitis but also in epithelial cells and goblet cells. Lee et al (33) found that apical TLR9 stimulation conferred tolerance to subsequent TLR challenge. Due to TLR9-assoiated activation, antigen-presenting cells produce IFN-α, resulting in activated Th1 cells, which produce pro-inflammatory cytokines, such as interleukin 6 and TNF-α. These pro-inflammatory cytokines activate neutrophil granulocytes, increasing the activity of MPO (34). The positive correlation between TLR9 expression and MPO activity detected in our experiment is consistent with a previous study (35). Our data, along with these observations, suggested that TLR9 plays a crucial role in maintaining intestinal homeostasis.

Studies by Sadeghi et al and Dickie et al (36,37) both reported that 1,25(OH)D_3_ suppressed the expression of TLR proteins and mRNA in human monocytes in a time- and dose-dependent fashion in vitro and reduced the recognition of bacterial specific antigens through monocytes. Another recent study showed that it decreased TLR-induced cytokine production and enhanced cytokine levels in peripheral mononuclear cells and monocyte-derived dendritic cells from CD patients (38). Our data showed that treating TNBS-induced colitis with 1,25(OH)D_3_ could reduce TLR9 expression in the rat colon. Moreover, we demonstrated that the severity of colitis was correlated with TLR9 expression. Thus, we hypothesize that 1,25(OH)D_3_ could relieve inflammation in TNBS-induced colitis by reducing TLR9 expression.

One of the study limitations is the small sample size, however the heterogeneity among rats was very low. We also did not use various concentration gradients of 1,25(OH)D_3_. According to the United States Institute of Medicine, the recommended dietary allowance of vitamin D in adult people is 15 μg/d (39). Different doses of supplemental vitamin D are based on the 1,25(OH)D concentration. Patients suffering from osteoporosis are usually treated with calcitriol at a dose of 1 μg/d. Thus, we used 0.2 μg/kg/d 1,25(OH)D_3_ to treat TNBS induced colitis, which is equivalent to 1 μg/d in a human subject. Finally, we only used TNBS, instead of dextran sulfate sodium, to induce IBD in rats. Our choice was based on previous studies, which showed that the TNBS-induced acute colitis is a Th1-mediated colitis, which resembles CD.

As vitamin D supplementation is readily available at a low cost, it is potentially a very attractive therapeutic option. The vitamin D analog, 22-ene-25-oxa-vitamin D (ZK156979) could both preserve immunosuppressive activity in vitro and ameliorate inflammation in TNBS-induced colitis without causing hypercalcemia (40,41), which offers a new therapeutic option for the treatment of human IBD. In conclusion, our study showed that 1,25(OH)D_3_ relieved TNBS-induced colitis in rats by down-regulating *TLR*-9, which might be associated with inflammation. Further studies are needed to clarify the exact molecular mechanisms involved in this process.
